# A reliable and robust online validation method for creating a novel 3D Affective Virtual Environment and Event Library (AVEL)

**DOI:** 10.1371/journal.pone.0278065

**Published:** 2023-04-13

**Authors:** Ifigeneia Mavridou, Emili Balaguer-Ballester, Charles Nduka, Ellen Seiss

**Affiliations:** 1 Centre of Digital Entertainment, Bournemouth University, Bournemouth, United Kingdom; 2 Emteq Labs, Sussex Innovation Centre, Brighton, United Kingdom; 3 Department of Computing and Informatics, Faculty of Science and Technology and Interdisciplinary Neuroscience Research Centre, Bournemouth University, Poole, United Kingdom; 4 Bernstein Center for Computational Neuroscience Heidelberg-Mannheim, Medical Faculty of Mannheim and Heidelberg University, Mannheim, Germany; 5 Department of Psychology, Faculty of Science and Technology and Interdisciplinary Neuroscience Research Centre, Bournemouth University, Bournemouth, United Kingdom; Justus Liebig Universitat Giessen, GERMANY

## Abstract

This paper describes the development and validation of 3D Affective Virtual environments and Event Library (AVEL) for affect induction in Virtual Reality (VR) settings with an online survey; a cost-effective method for remote stimuli validation which has not been sufficiently explored. Three virtual office-replica environments were designed to induce negative, neutral and positive valence. Each virtual environment also had several affect inducing events/objects. The environments were validated using an online survey containing videos of the virtual environments and pictures of the events/objects. They survey was conducted with 67 participants. Participants were instructed to rate their perceived levels of valence and arousal for each virtual environment (VE), and separately for each event/object. They also rated their perceived levels of presence for each VE, and they were asked how well they remembered the events/objects presented in each VE. Finally, an alexithymia questionnaire was administered at the end of the survey. User ratings were analysed and successfully validated the expected affect and presence levels of each VE and affect ratings for each event/object. Our results demonstrate the effectiveness of the online validation of VE material in affective and cognitive neuroscience and wider research settings as a good scientific practice for future affect induction VR studies.

## Introduction

Virtual Reality (VR) offers a flexible tool to construct affect evoking situations or environments which can be used in addition to more traditional methods such as the usage of popular affective images or videos libraries (e.g., IAPS [1]). For long, we have theorised that this tool might be more powerful than more conventional passive formats that are commonly used in affect provoking studies. More specifically, the change of the medium from still images to videos has enhanced the potency of the affective experience, and thus increased the intensity of the behavioural and physiological responses of the observer. This can be attributed to changes in medium properties such as the screen size, colour, motion and audio incorporation; all of which increase ecological validity [2–6]. Immersive VR technologies offer an even higher degree of ecological validity due to high-resolution display properties, audio incorporation, high frame rate for movement-eye synchronisation and, importantly, the due to the possibility to interact with features in the virtual space. These VR features can even adapt based on the behaviour and actions of the user.

The biggest benefit of using VR for affect research, is that it allows the creation of more real-like and naturalistic affective experiences in highly immersive interactive settings which can evoke naturalistic affective and behavioural responses [7–9]. In fact, it was found that higher levels of immersion and presence can induce significantly higher self-reported arousal responses [10, 11] and more intense emotions [12–14]. It was shown that one of the main differences between non-immersive and fully-immersive VR experiences is the level of presence they can elicit to the user (presence is the sense of being in a virtual environment rather than the place in which the body is physically located [15–17]). Presence is positively related to the interaction of user with the content and to the coherence of body actions with spatial VE structure [15, 18–21]. Modern VR technologies allow for interactivity with the content, freedom to explore spatial elements, and contextual synthesis of the scenarios. All of this creates a stronger sense of plausibility, which is not leveraged with predominantly passive 2D content. As such we need to explore VR as more than a passive presentation or simulation medium and use its potential as an immersive, interactive, and ecologically valid medium that can induce different levels of affect and be experimentally controlled at the same time.

However, existing stimulus libraries for emotion elicitation using interactive immersive VR content is scarce. Recently two affective 360° video libraries were developed for use with immersive technologies and validated using surveys. The first library published in 2017, contains 360° video-stimuli which can be passively experienced in VR settings [22]. The second library is called “luVRe” and it contains pre-recorded 3D-360 videos of everyday scenes [23]. Both datasets open new avenues for controlled affect induction research in passive observing immersive settings. However, pre-recorded video datasets are inherently less interactive and less adaptive than interactive VEs. In the last couple of years, few 3D object libraries were published. Amongst them were the “virtual objects dataset” with 121 validated items [24], a “3D standardised for Virtual Reality” library with 147 items [25], and the “OpenVirtualObjects” library [26] offering a set of 124 realistic 3D household objects. However, the above object libraries were validated for characteristics such as name agreement familiarity, and visual complexity, but not for affective ratings. This year, a new interactive content library was published byby Dozio [27, 28], in which VE environments were designed to elicit different intensities of five discrete emotions and ranges of affect. Albeit good evidence of the efficiency of the proposed VEs to provoke the targeted emotions, the individual affective attributes of the visual and acoustic elements pertaining each VE were not individually rated. As such, specific event / object manipulations to improve the efficiency of the entire VE could be missed.

The new interactive content library by Dozio [27, 28] used one of the two most common approaches of modelling affective change which is the discrete emotion model. This first model is heavily rooted in distinct emotion state detection, such as the ‘basic emotions’ [29–35], which are complex, culturally dependent, highly subjective experience, which are difficult to be broken down to smaller parts. Regardless of its straight-forward nature, this model is not always preferred for multimodal affective computing research, due to its strict, deterministic structure, and the complexity to identify and distinguish some states from others via physiological data [36]. This model was also recently heavily criticised in the affective neuroscience literature [37] and it has been shown that physiological and neuroscience measures are often not able to clearly distinguish specific emotions. By comparison, the second approach to measure affective change uses the dimensional model of affect [38] which contains primarily the dimensions of valence and arousal. This model does not only relate to other models including the conceptual act model [39], but it is are supported by recent neuroscientific evidence [37]. Affect is the core function of emotional responses, the energy flow that precedes and produces those altogether experiences. In the dimensional model, physiological and psychological changes can be measures with valence (positive to neutral to negative), and an arousal intensity (low to high). Being aware of the debate between discrete and dimensional models of affect, our team decided to mostly build their research on valence and arousal detection methodologies and metrics, with the ultimate aim of combining metrics with continuous physiological measures in VR settings.

To our best of knowledge there is no truly interactive VE library for affect induction that uses standardised validation protocols for the entire virtual environments and key events/objects within the virtual environment. This article gives a clear example about how this VE library could be built and achieved in a standardised manner by using online surveys for the VE validation in a first instance before the VE can be re-validated in more time consuming and expensive experimental laboratory-based studies. This initial step online survey also allows the testing of more options before the laboratory-based study is conducted. For the reported example, several interactive room-scale virtual scenarios were designed and developed, giving us full creative and experimental control over the properties of these VEs. The VEs induced different levels of valence and arousal states. Valence and arousal are the two core measures of affect according to the dimensional model of core affect [38]. Valence describes the range of an affective state from negative to neutral to positive. Arousal describes the physiological intensity that can range from low (sleepy/calm) to high (alert/excited). All room-based VEs were identical, except for some specific events/objects placed into them. Some of these events could be triggered in an interactive fashion. The ability of those environments and their elements to induce the targeted affective impact was validated using an online screen-based survey. As stated above, this online survey constitutes of a time- and cost-effective pre-VR validation approach for the audio-visual elements pertaining the VEs, thus allowing for redesign or adjustment prior to running long and expensive VR experiments.

The aim of this study was to validate the novel purpose-built affective 3D VE library in an extensive online survey study. Our primary hypothesis was that the affective VEs, and the specific events/objects within these VEs, can elicit the expected affective levels of valence and arousal (Negative VE: high arousal, negative valence; Positive VE: high arousal positive valence; Neutral VE: low arousal, neutral valence, Baseline VE: low arousal, neutral valence). Secondarily, as a manipulation check, we hypothesised that the memory accuracy is enhanced for more affective events/objects than non-affective ones because they require deeper levels of processing. In addition, the most memorable events within each condition are also the ones eliciting high affective arousal rating. This was shown in previous work with conventional (i.e., 2D) stimuli where emotionally arousing stimuli were more likely to be memorized and recalled compared to neutral stimuli [40–43]. Thirdly, we predicted that affective VEs also had higher presence rating. Finally, we hypothesized that affective valence and arousal ratings are reduced in people with high alexithymia levels because it is inherent to this condition to have a deficiency in identifying and processing emotional information [44].

## Participants

The online validation survey was filled out by 91 participants. As part of the survey, participants were screened for severe psychological, mental disorders and/or encephalopathy. Participants who self-reported severe phobias of spiders (arachnophobia), fires (pyrophobia), enclosed spaces (claustrophobia) and fear of the dark (nyctophobia) were excluded from the survey (n = 22). Another two participants were excluded due to invariant responses.

The final data set for data analysis was 67 participants (62 females, 5 males; mean age: 19.78 years (SD: ± 2.51 years)). In terms of experience with games, 7.5% had no experience, 58.5% had little experience (novice), 30.3% had average experience with games, and 13.6% considered themselves as experts. Participants had little experience with virtual reality overall, with 36.3% having no experience at all, 43.9% having little experience, and 19.7% having average experience. None of the participants considered themselves experts with VR. The participants were recruited either at the Sussex Innovation Centre and included staff members, professionals and academics) or from the student population at Bournemouth University via online advertisement. The study was reviewed and approved by the Research Ethics Panel of Bournemouth University (reference ID: 18848). The participants were compensated for their time with SONA research credits (students only) or Amazon vouchers (£5).

## Materials

### The virtual environments (VE)

The software used for the development of the VE scenarios was Unity3D game engine [19], and the software used for the design of the 3D events/objects was Autodesk Maya 3D [20].

**Fig 1:**
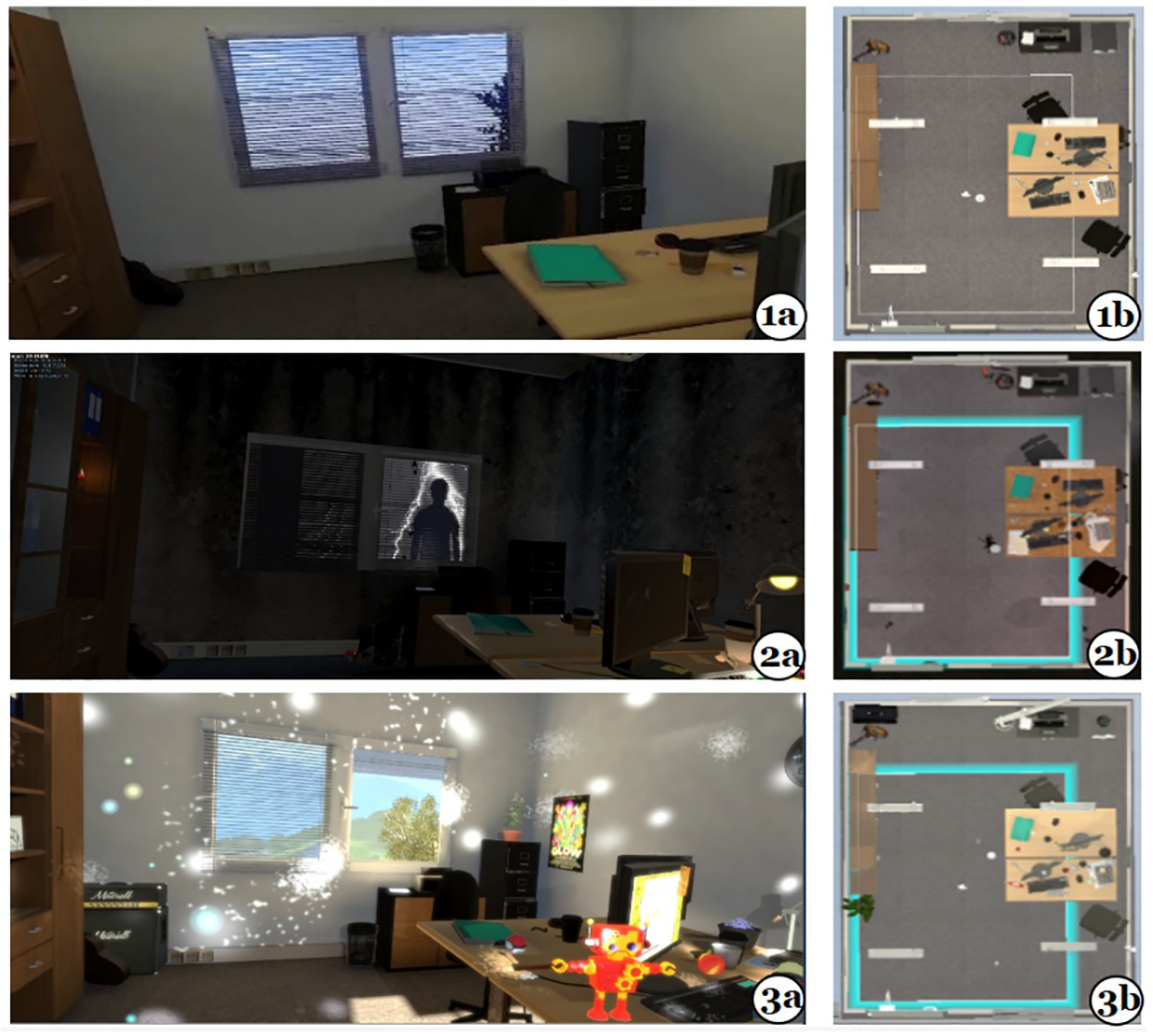
Screenshots of the three affective VEs from the user’s view (a, left side) and top view (b, right side). 1a & 1b (top): Neutral VE; 2a & 2b (middle): Negative VE; 3a &3b (bottom): Positive VE.

Following the dimensional model of affect approach [38] we created four separate VEs (a neutral, a negative, a positive, and a baseline VE). The neutral, negative and positive VEs were exact replicas of an office room and all three shared the exact same spatial dimensions and structural architecture (see Fig 1). The virtual office room was 2.3m width x 2.90m length x 2.20m height with an allocated walking area of 1.6m x 2m which was consistent between all VEs. The VEs were mapped according to that space and populated with virtual counterparts of the physical objects. In all settings, the room contained a bookcase, two office desks with chairs, a window, lights, two PCs with monitors, a small cupboard with a printer, a garbage bin, a mirror and two paper notebooks.

The office-room replicas were then decorated and processed to convey the targeted valence ranges. Existing literature on the effect of low-level audio-visual features (e.g., brightness, colour hue, sound manipulations) informed the design of the VEs [45–48]. The environments were populated with 3D objects and events which were designed to enhance the overall targeted affective impact for each VE and to induce different variations of arousal. The objects and events had various sizes and attributes. They were placed within the virtual rooms, in various locations, taking advantage of the overall virtual space and structure of the room replicas, thus allowing the user to experience them by freely exploring the rooms (room-scale interaction). Some of those stimuli were static objects while others were animated events (activated by gaze-interaction) with audio and visual elements. The created office scenes are shown in Fig 1. The events and objects used for each scene are listed in Table 1.

**Table 1:** List of objects and events for each office VE.

	Negative VE	Neutral VE	Positive VE
1	Fire Alarm	Bookcase	Green plant
2	Documents	Clock	Baby poster
3	Window -Lightening/silhouette	Green Notebook	Light explosion
4	Glitch in viewpoint	Grey Notebook	Reflection mirror
5	Fire	Guitar	Dog poster
6	Overflowing bin	Window	Butterflies
7	Flickering light (bulb fusing)	News board	Robot
8	Spooky mirror	Calendar/Cup	Monitor message
9	Spiders in room	Computer Mouse	Stardust (Light particles)
10	Light (bulb exploding)	Desks	Guitar
11	Spilt drink (cup)	Bin	Flower
12	Rat	Mirror	Birds
13	Spider attack	Carpet Floor	Amplifier
14	Spooky music	Monitor	Beach ball

#### Neutral VE

The neutral environment contained all static basic objects of the office synthesis without the elements that were designed for negative or positive valence elicitation. The colour palette and temperature were kept in grey and cold faded tones, with low contrast to reduce the possibility for increased physiological arousal [21, 49]. No audio samples or interactive events were used in this VE. The lighting conditions were soft and dimmed with smooth shadows. This way the room was not strongly lighted nor bright, but completely visible for exploration by the user. A roman blind was designed in front of the window, to reduce the incoming light from the virtual sun embedded in the scene. The space outside the room, visible through the window was set to a grey, cloudy view with faded colour detail.

#### Negative VE

The colour palette for this room was set to intense, dramatic contrasts. The overall atmosphere was inspired from two horror games in VR: ‘Resident Evil 7 VR’ and ‘Affected: the Manor’ [50, 51]. The main lights were direct, switching on and off as if they were faulty using a custom-built switch with short, randomised time intervals. The walls and floor were covered with an additional material resembling of dirty, unpainted concrete. The synthesis of lights and textures was based on the design experience of the research team. Multiple objects were placed inside the room, including interactive and animated spiders, stressful notes, an animated ghost-like figure in the mirror, an animated shadow-figure appearing outside the window, an animated rat, a candle, litter placed around the bin, a fire event and an alarm. The majority of these events were triggered by the gaze of the user, e.g., ghost figure in the mirror. Others like the crawling spiders were activated throughout the whole VE experience by slowly follow the users gaze in the room, climbing on walls and main virtual furniture. The rest of the events, such as the ‘spider attack’ event, were triggered once, based on time, attempting to jump-scare the user. For example, once the user had spent 65 seconds in the scene, a fire was triggered, and 10 seconds later the fire alarm went off requesting the user to head towards the exit and leave the room. The fire alarm included a bright, red light circling around the room in quick intervals and a loud siren. These events were designed to increase the physiological arousal towards the end of the experience and intensify the negativity of valence. Audio were incorporated in all interactive objects, including light bulbs, the ghost event, the shadow (lightning), the rat, the fire alarm, and the spiders. Some of audio were downloaded from the free sound audio library [52].

#### Positive VE

Similarly, in this positive VE, audio-visual parameters were set to provoke pleasant feelings with variations of arousal for the positive environment. Multicolour synthesis was selected, including bright tones with intense colour hues and saturation. All lights were set brighter across the whole room and an intense sun light was designed to enter through the window. The roman blinds were not covering the view anymore allowing for the user to look outside the window. Along with static objects including posters, post-its, an apple, and pictures, multiple interactive events were programmed to be activated in different times based on the gaze of the user. These interactive events included a flock of butterflies entering the room through the window, birds flying outside the window, fairy lights or star dust inside the room, a robot dancing on the table, webcam feed of the user on the mirror, flowers growing, and a plant moving. Again, audio was incorporated in all interactive events and animated objects. After about 75 seconds in the room, and if the user was not engaged with another event/object, the windows would open allowing laughter sounds to enter the room.

#### Baseline-relaxation VE

**Fig 2:**
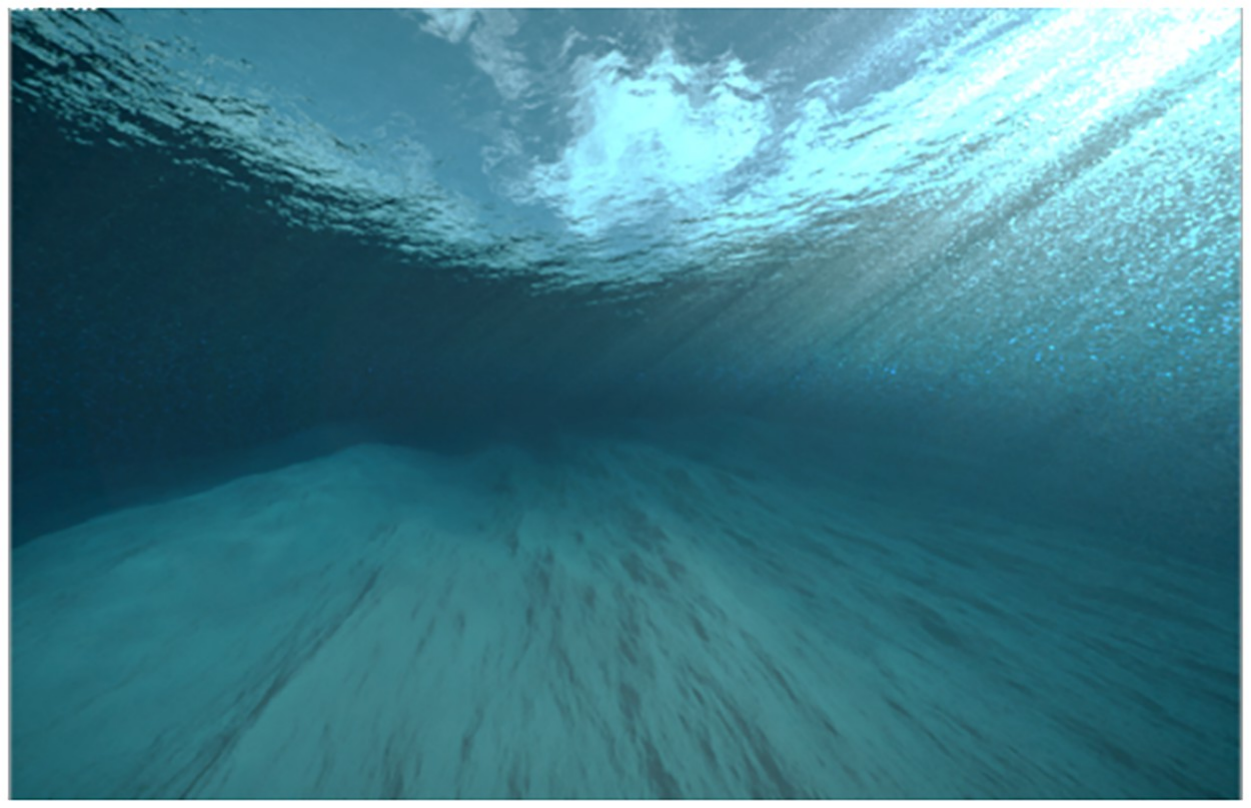
Screenshot of the baseline VE condition as used in the online survey.

A fourth environment was added as a baseline condition to record physiological responses before entering the three office VEs. A 360° underwater environment with a smooth water audio-track (Fig 2) was used. Aquatic elements and marine biota were not added to avoid high positive valence ratings [53]. The introduction of the water element of this VE was inspired by research on the restorative effects of aquatic environments [54, 55]. We expected this environment to evoke low arousal and neutral/slightly positive valence levels.

### Videos & images from the VEs for the online survey

For each VE, a walk-through video (duration: 76 seconds) was embedded in the survey. Each video was recorded from the point-of-view of a VR-user taking part in the pre-pilot of the VR study. After each VE video, short videos and images of individual events were presented in the online survey (14 events per VE). The fourteen events per VE scenario (neutral, positive, negative) were extracted as images and videos giving a total of 42 object-items to be rated; videos were preferred for interactive or animated objects/events involving sounds, and still images were preferred for static objects. Table 1 shows the list of events and objects per VE scenario. Screenshots of all events and objects are available in the supporting materials link.

### Questionnaires and ratings used in the online survey

The demographic questionnaire included questions on age, gender and experience with immersive technologies.

The Toronto Alexithymia Scale (TAS, 20 items) was used [56] because high alexithymia is a trait related to the reduced ability of an individual to identify and describe emotions of their own and others, while also as a deficit on regulating affect [57]. Responses were provided using a 5-point scale ranging from 1 (strongly disagree) to 5 (strongly agree).

The IGroup Presence (IPG) questionnaire [18, 58] was used to measure the level of presence induced by the VEs. The questionnaire consisted of 14 items, for example, “I was completely captivated by the virtual world”. The IPQ is a popular questionnaire in VR research [59, 60] as it has exhibited good psychometric properties [61]. Responses are provided using a 7-point Likert scale [45] ranging from -3 (not at all) to 3 (very much). These responses were then mapped to 1 to 7 for analysis purposes. The questionnaire consists of three independent subscales, plus a general presence item not belonging to any of the subscale. The subscales are namely ‘spatial presence’ (the sense of physically being present in the VE), ‘involvement’ (the level of devoted attention and involvement in the VE) and ‘experience realism’ (measuring the subjective experience of realism in the VE) [34]. The additional item assesses the general feeling of presence as the ‘sense of being’ in the VE.

The valence and arousal levels were measured using self-assessment manikins (SAM) figures [62] and visual analog rating scales [63] beneath these SAM figures. The scale used for valence ranged from 1-very negative (unpleasant) to 9-very positive (pleasant). The scale used for arousal ranged from 1 low (sleepy) to 9 high (active, excited).

A 1-item memory accuracy question was added for each event/object in the online survey. The question asked if the participants remembered this event or object (‘yes’ or ‘no’).

### Procedure of the online survey

The online survey was programmed using Qualtrics Software [64]. The participants were instructed to use a PC or laptop screen for the completion of the survey. It was made tablet and web-browser ready for remote viewing. It consisted of four parts. The first part included a participant information sheet with the description of the study and the used methods, as well as the consent form. This part also included the short demographic questionnaire.

The second part was a practical instruction to ensure that participants were familiar with the format of the survey and the meaning of the valence and arousal rating scales. Examples were given using videos which were not the to be validated VEs. The participants were reminded that the rating of each video or picture should reflect their immediate personal experience. Then, a brief sound check was performed using a short bell sound. This ensured that all users could hear the auditory information of the videos. For this point onward, participants were instructed to not switch or alter their brightness and volume levels anymore. The participants were encouraged to relax before starting the survey and were reminded to take short breaks of up to 5 minutes throughout the survey.

The third part was divided into four sections. The four sections were dedicated to the validation of each of the four created VEs’ videos, i.e., baseline, neutral, positive, and negative VE. These sections were counterbalanced across participants. Participants were instructed to imagine their experience as if they were within the presented VE when watching the video. Time-based control was added to the videos to ensure the participants had watched the whole video before rating it. After each video presentation, participants provided subjective valence and arousal ratings. With the exclusion of the baseline VE, each video was followed by the presentation of fourteen events presented previously in the VE video whose sequence was randomised. Participants rated the valence and arousal levels of each event/object and answered the memory question. Once the VE-video and corresponding events were rated, participants were asked to rate their perceived feelings of presence with the IGroup Presence questionnaire. Here, the option to view the video of the VE again was given to help them to recall the initial feeling presence.

In the fourth part, participants were asked to complete the TAS. The survey took approximately 1 hour.

## Results

The results are divided into five sections: (a) results for each VE condition (positive, neutral, negative) when analysing the ‘VE video’, (b) results for each VE condition when analysing event-related measures relative to the occurrence of static and interactive objects, (c) memory accuracies for each VE, d) presence ratings for each VE, and e) individual differences in alexithymia ratings and their relation to arousal and valence ratings in each VE environment. The findings were presented by following the guidelines set by [65].

### Affect elicitation reliability of entire VE

**Fig 3:**
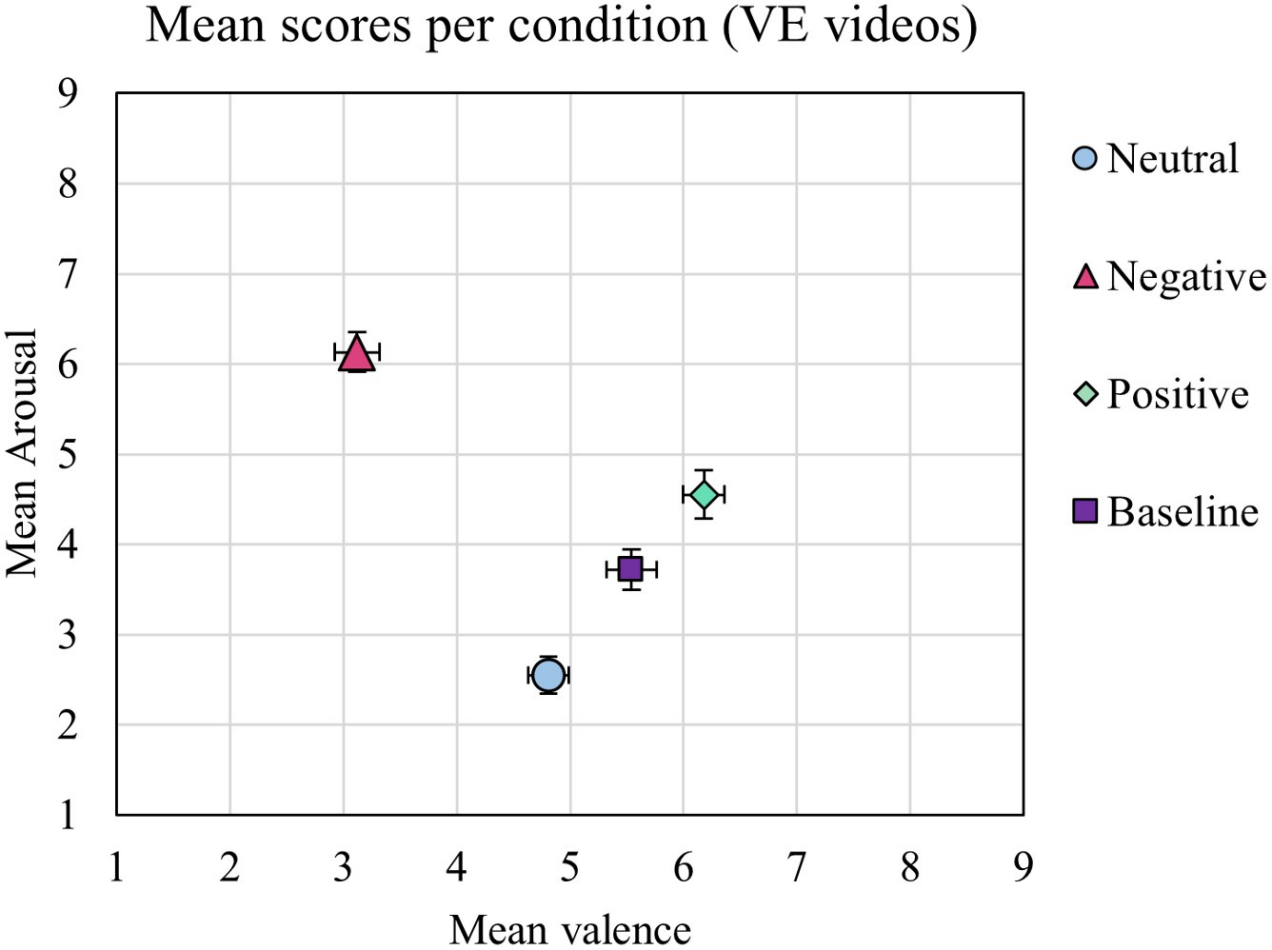
Mean valence and arousal ratings for each VE condition: Neutral VE (light blue circle), Negative VE (pink triangle), Positive VE (green diamond), Baseline VE (purple square). Error bars depict SE for each dimension.

First, the affective impact in terms of valence and arousal was assessed for each VE video. The mean arousal and valence ratings were calculated for three office-based VEs (neutral, positive, negative VE) and for the underwater-themed relaxation-baseline VE. These mean ratings are presented on the affective space diagram in Fig 3. As expected, the neutral VE was rated as neutral in valence (Mean: 4.81, SD: ±1.47) and a low in arousal (2.55 ±1.67). The ratings for the negative VE were low in valence s (3.12 ±1.64), meaning they were perceived as negative, and high in arousal levels (6.13 ±1.81). The ratings for the positive VE were high in valence (6.18 ±1.49), meaning they were perceived as positive, and moderately high in arousal levels (4.55 ±2.21). The baseline VE generated for valence ratings close to neutral (5.54 ±1.83) and low arousal ratings (3.72 ±2.24).

#### Inter-Rater Agreement for VE videos

The coefficient of variation (CV = SD/mean) was used as a measure of calculation of the dispersion of the participant’s ratings for each video. Two CVs were calculated as percentages for each VE, one per affective dimension. Low CV shows low dispersion and therefore high agreement between raters. Regarding the valence ratings, the positive, neutral and baseline videos were rated in high agreement between participants (CVval_Pos = 24%, CVval_Neutral = 30.5%, CVal_Baseline = 33.07%) for their perceived valence (CV < 50%, with criteria of 0.50%). However, the negative video generated higher interrater variability for the valence scores across participants (CVval_Neg = 52.7%). The inter-rater agreement for the arousal scores were as follows. Raters had a good agreement for the positive video (CVar_Pos = 48.5%), and a high agreement for the negative video (CVar_Neg = 29.5%) The arousal ratings for the neutral video varied more considerably across participants (CVar_Neu = 65.4%). This was also the case for the baseline video (CVar_Base = 60.22%).

#### Valence ratings of VE-videos

In line with our hypothesis, valence ratings in the negative VE video were low and valence ratings in the positive VE video were high. Valence ratings for the neutral and baseline VE scenes were intermediate. A Shapiro-Wilk test of normality was performed and indicated that ratings for both dimensions were not normally distributed (p < .05). Therefore, a non-parametric Friedman’s test was conducted on the valence ratings for the four VE conditions. The test showed that the ratings were significantly different between VE conditions, *χ*^2^(3) = 73.85, *p* < .001. Consequently, Bonferroni corrected post-hoc Wilcoxon tests were conducted. These tests showed significant differences between most conditions. More specifically, the valence ratings in the negative VE were significantly lower than in the neutral VE (*z* = 5.39, *p* < .001), the positive VE (*z* = 6.77, *p* < .001, r = .83), and the baseline VE (*z* = 6.27, *p* < .001, r = .77). Valence ratings for the positive VE were significantly higher compared to the neutral VE (*z* = 4.706, *p* < .001, r = .57), but not compared to the baseline VE. The baseline VE had also significantly higher valence ratings than the neutral VE (*z* = 2.62, *p* = .036, r = .32). These findings show that the three office-based VEs achieved expected valence ratings. However, the baseline VE room induced more positive valence ratings than intended.

#### Arousal ratings of VE-videos

A Shapiro-Wilk test of normality indicated that ratings for both dimensions were not normally distributed, and thus a non-parametric Friedman’s test was conducted on the arousal ratings for the four VE conditions. The test showed significant differences between the four VEs, *χ*^*2*^(3) = 79.86, *p* < .001. Bonferroni corrected pairwise post-hoc Wilcoxon tests showed significant differences between arousal scores reported. More specifically, the arousal scores for the negative VE were significantly higher than for the neutral VE (*z* = 6.78, *p* < .001), the baseline VE (*z* = 5.59, *p* < .001, r = .68), and the positive VE (*z* = 4.81, *p* < .001, r = .59), rendering the negative VE as the most arousing condition. The positive VE had significantly higher arousal ratings compared to the neutral VE (*z* = 5.42, *p* < .001, r = .66), and the baseline VE (*z* = 2.31, *p* = .021, r = .28). Unexpectedly, the baseline VE achieved a significantly higher arousal ratings than the neutral VE scene (*z* = 3.91, *p* < .001, r = .48). In summary, the three office-based VEs had expected mean arousal ratings. However, the baseline VE had higher arousal ratings than intended.

The results from the statistical analysis showed that the valence and arousal ratings of the VE videos were significantly different from each other, with the neutral scene being rated as neutral in valence with low arousal levels, while the affective scenes (positive and negative) were able to elicit stronger affective states in terms of higher arousal and strongly antithetical valence levels in the Arousal-Valence (AV) space. However, the aquatic baseline scene did not portrait our initial design expectations, scoring higher in valence and in arousal ratings than the neutral scene.

### Event-related reliability analysis

#### Valence & arousal ratings

**Fig 4:**
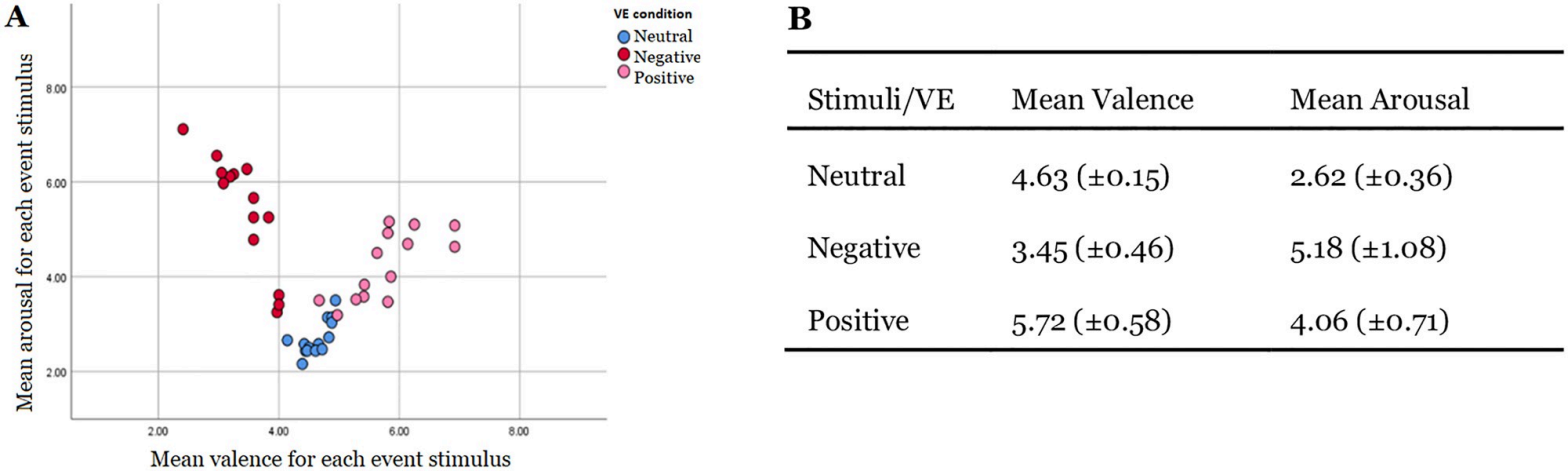
Panel A: Mean valence and arousal ratings for each event/object presented in the three office-based VE conditions. The x-axis (bottom) displays valence ratings and the y-axis (left) displays arousal ratings. Panel B: Mean valence and arousal scores averaged across all events/objects for each VE.

Mean valence and arousal ratings for each event/object are displayed in an AV space format in Fig 4. The depicted ‘V’ shape (panel A) of ratings is similar to valence and arousal ratings reported in several other studies [66]. In panel B the mean valence and arousal ratings averaged across events/objects are presented for each VE separately (±SD).

#### Inter-rater agreement

The coefficient of Variation (CV) was computed for each event/object from the arousal and valence ratings for all participants. Overall, low inter participant variation (high agreement) was found for valence ratings in all three VE conditions (mean_CVval < 50%). This was also the case for arousal ratings (mean_CVar < 55%). Saying that, the participant agreement was a somewhat lower for arousal ratings compared to the valence ratings, as reflected in higher CV scores. More specifically, high agreement was recorded for valence ratings in the positive condition (30.92±2.97%). The variation was slightly higher for the valence ratings in the negative condition (49.99±10.10%). For the arousal ratings, the highest agreement was present in the negative condition (41.88±8.54%), followed by the positive condition (53.4±5.56), and the neutral condition (63.4±3.06%). The CV results per event can be found in the supporting information.

The CV agreement ratings based on the mean calculated across all the events per VE followed the same pattern as the CV agreement ratings per VE. For both, high inter-rater agreement was observed for positive, neutral valence but not for negative valence ratings. For arousal ratings, higher-interrater agreement was found the negative VE compared to the other two conditions.

### Memory accuracy correlated with affective intensity

As a first step, we calculated the mean memory accuracy scores averaged across all events per VE condition. The mean accuracy across events was for the neutral condition 71.12±39.02%, for the negative condition 68.66±36.20%, and for positive condition 76.33±37.80%. These results showed an overall high average level of memory accuracy for all three scenes. However, mean memory scores across events did not differ significantly between VEs (Friedman test: *χ*^2^(2) = .143, *p* = .931), showing that all three conditions were equally memorable. Note, the variability of the answers was high in this online survey.

**Fig 5:**
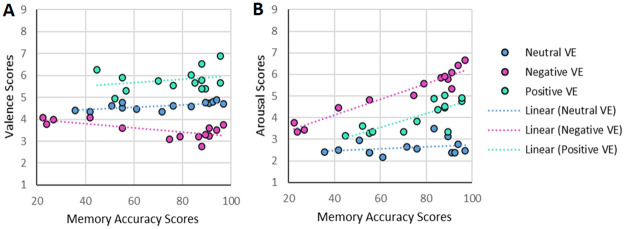
Panel A—relationship between valence ratings (y-axis) and memory accuracy for each event/object. Panel B—relationship between arousal rating (y-axis) and memory accuracy (x-axis) for each event/object. Red dots represent events/objects from the negative VE condition, green dots represent events/objects from the positive VE condition, and blue dots represent events/objects from the neutral VE condition.

Then, based on the hypothesis that memory accuracy is enhanced for more affective stimuli, Spearman correlations between the mean memory accuracy scores across all events and the valence and arousal ratings were analysed, separately for the positive and negative VE condition. The findings showed that positive or negative valence ratings and higher arousal ratings are strongly correlated with higher memory accuracy (Table 2). The correlations were higher for negative compared to positive VEs. Please note, memory accuracy is increasing with simultaneous increase of valence and arousal ratings (see Fig 5), therefore decoupling of valence versus arousal effects on memory accuracy is difficult.

**Table 2:** Correlations between memory accuracy and mean valence and arousal ratings across events/objects for the positive and negative VE conditions.

Memory scores *	Corr. Coef. (r)	P (Sig)
Valence (Negative VE)	-.86*	< .001
Arousal (Negative VE)	.92*	< .001
Valence (Positive VE)	.56*	.039
Arousal (Positive VE)	.68*	.007

*Significance at a 0.01 level

Afterwards, mean memory accuracy scores were also calculated separately for each object/event and for each VE condition (see S1 Table in S1 Data) in order to analyse more event/object specific information. For this, mean memory accuracies were examined for each event/object in reference to their corresponding valence and arousal ratings. From the event/objects used in the negative condition, the ‘spider attack’ event was the most memorable, which was also the most arousing, event (Vscore = 3.76, Ascore = 6.66), followed by the ‘mirror’ (ghost figure) event (Vscore = 3.52, Ascore = 6.43), and ‘spiders’ across the room (Vscore = 2.76, Ascore = 5.90). Similarly, the most memorable stimuli for the positive scene were the ‘butterflies’, the ‘robot’ and the ‘dog poster’, whereas, the least memorable stimuli were ‘guitar’,’ flower’ and ‘ball’. Not surprisingly, the least memorable objects/events across all three VE conditions were the static objects, such as the ‘cup’, ‘guitar’, ‘documents’, ‘grey notebook’, and ‘rubbish bin’ (mean memorability <50%). Although they existed in all scenes, they were not expected to be memorable nor arousing. The remaining objects and events rendered a high memorability (between 55–95%), with the exception of the ‘rat’ event in the negative scenario which scored lower than expected. The low memory accuracy score could be caused by the reduced visibility of the “rat” in the video used in the survey (located on the lower bottom of the screen).

### Affective VE content enhances presence

The IGroup Presence questionnaire [34] was analysed for the three affective VE conditions (positive, neutral, negative). As expected, all three VE conditions had low presence scores because the VE experiences were presented as videos through non-immersive interfaces in an online survey. The mean presence score for the neutral condition was 1.77±1.02, for the positive condition 1.89±1.13, and for the negative condition 2.62±0.55. These presence ratings significantly differed between the three VE scenarios (Friedman test: *χ*^2^(2) = 34.17, *p* < .001). Following up on this, Wilcoxon tests with Bonferroni corrections indicated significant differences between the neutral and negative conditions (*z* = 5.55, *p* < .001, r = .68) and the positive and negative conditions (*z* = -5.12, *p* < .001, r = .63), but not between the positive and neutral conditions (p>.05). This signified that the negative VE condition elicited significantly higher feeling of presence than the other two VE conditions.

**Fig 6:**
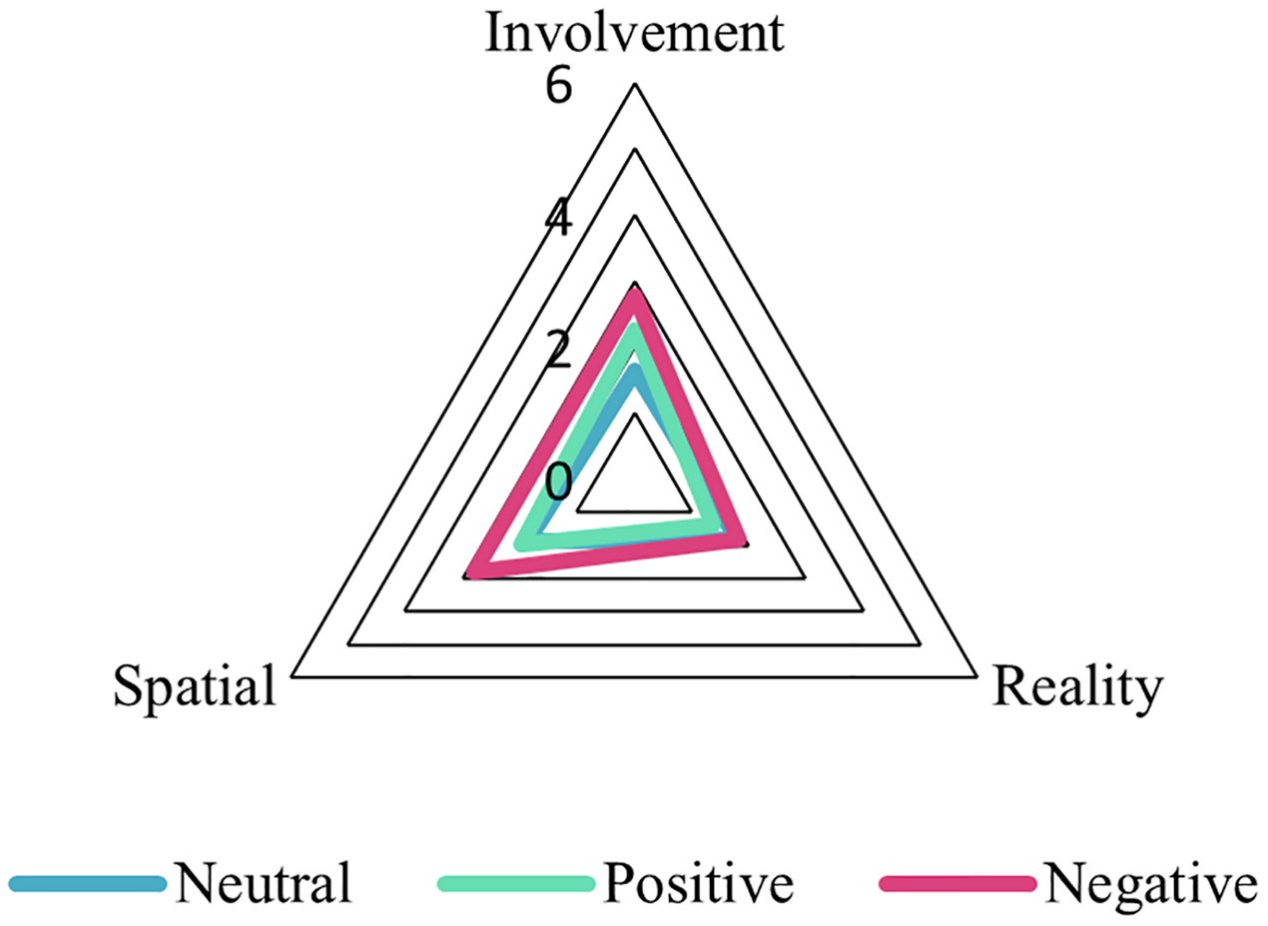
Radar plot showing average scores for each subscale: Involvement, spatial presence (‘Spatial’) and experience realism (‘Reality’) per VE. General presence is excluded. The surface generated from the subscales contributes to the average presence felt for each VE (Neutral (blue outline), Positive (green outline), Negative (pink outline). The negative VE shows greater presence than the other VEs.

For each VE, presence scores were also calculated separately for the subscales of spatial presence, involvement, and experience realism, and for the general presence item. These mean presence scores are displayed in Table 3. In addition, these mean presence scores are shown in a radar plot for better visualisation in Fig 6. Again, presence scores were higher for the negative VE condition compared to the other VE conditions for the spatial presence and involvement subscales, and for the general presence item. An exception is the experience realism subscale where presence scores were lowest for the positive condition and similar for the negative and neutral conditions.

**Table 3:** IGroup presence scores (±SD) for each subscale and VE condition.

	Neutral VE	Positive VE	Negative VE
General Presence	1.75(±1.72)	1.94(±1.62)	3.06(±1.74)
Spatial Presence	1.87(±1.24)	1.96(±1.33)	2.80(±1.43)
Involvement	1.64(±1.21)	2.27(±1.25)	2.78(±1.43)
Experience realism	1.80(±1.11)	1.38(±1.09)	1.84(±1.20)

In order to analyse the link between presence ratings and affective ratings, presence ratings were correlated with arousal and valence ratings, separately for each VE condition. A significant positive correlation was revealed between the arousal ratings and the presence ratings for the negative VE (*r* = 0.362, *p* = .003) and for the positive VE *(r* = 0.257, *p* = .036), regardless of the polarity of the affective context. For valence, there was also a significant negative correlation between valence ratings and presence ratings for the negative VE condition *(r* = -.263, *p* = .031), and a trend towards a significant positive correlation for the positive VE condition (*r* = 0.24, *p* = .054). The results confirm the relationship between affective content and presence ratings. Arousing environments are related to enhanced presence independent of affective content. In addition, negative (and potentially positive) content is also related to an increased feeling of presence.

### Alexithymia effects on arousal and valence self-ratings

For the alexithymia questionnaire analysis, participants were divided into two groups based on their alexithymia score (division point = 61, as used in previous research [56]). Participants with alexithymia scores higher than ‘61’ were allocated to the high alexithymia group (N_h_ = 17) and the remaining participants were allocated to the low alexithymia group (N_l_ = 50).

Valence ratings of the entire VE video did not differ between alexithymia groups. This was confirmed with a 3 x 2 mixed ANOVA with the within-participant factor VE conditions (neutral, positive, negative) and the between-participant factor alexithymia group (low vs. high). The ANOVA revealed no significant main effect of the alexithymia group (*F*(1,65) = 1.89, *p* = .173, η^2^ =) and no interaction between alexithymia group and VE condition (*F*(2,130) = 2.59, *p* = 0.68, η^2^ = .68). For arousal ratings of the VE videos, again a 3x2 mixed ANOVA was conducted and it did not show any rating differences between alexithymia groups as reflected in the absent main effect of alexithymia group (F(1,65) = 1.90, p = .173, η^2 =^) and no significant interaction between the alexithymia group and the VE conditions(*F*(2,130) = 1.24, *p =* .253, η^2^ = 0.19).

**Fig 7:**
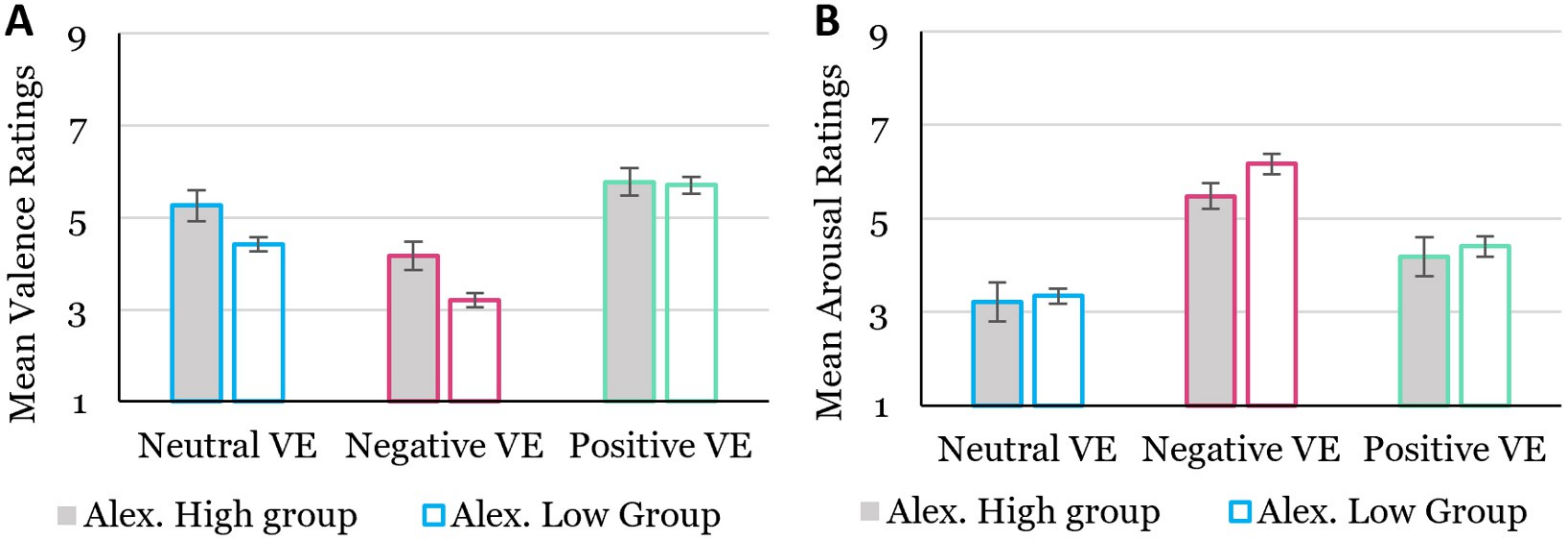
Mean valence and arousal ratings per alexithymia group. Left figure (A): Mean valence ratings across events for each condition. Right figure (B): Mean arousal ratings across events for each condition. (SE error bars).

As a second step, we computed the event-based average valence and arousal ratings across all events per VE. Fig 7 displays the mean valence ratings (left side) and mean arousal ratings (right side) for each alexithymia group and VE condition. As shown in Fig 7 (left side), The valence ratings were analysed as previously, but this time they revealed a significant main effect of the alexithymia group (*F*(1,65) = 5.83, *p* = .019, η^2^ = .082), as well as a significant interaction between alexithymia group and VE condition (F(2,130) = 3.26, p = .044, η^2^ = .48). Post-hoc analysis showed that the two alexithymia groups reported more neutral valence ratings (reflected in significantly higher valence scores), in the neutral and the negative VE (Neutral: High_A_ = 5.3, Low_A_ = 4.4; t(65) = 2.27, p = .033, d = .72, Negative: High_A_ = 4.2, Low_A_ = 3.2; t(65) = 2.80, p = .010, d = .85) but not in the positive VE (t(65) = .201, p = .837, d = .056). The ANOVA using the mean arousal ratings did not reveal a significant main effect for the alexithymia groups (*F*(1,65) = 1.86, *p* = .177, η^2^ = .028) and also no interaction between alexithymia group and VE condition (*F*(2,130) = 1.24, *p* = .294, η^2^ = .019). The results overall suggest that a more targeted, event-based analysis that concentrates on key events can show clearer effects in individual differences in alexithymia compared to an overall rating of the entire VE.

## Discussion

The capacity of VR as a technology to induce more vivid affective states in interactive environments whilst allowing the experimenter to have full control over the VE setup, makes it increasingly more common affective cognition and neuroscience [67, 68]. However, to the best of our knowledge, there is only one truly interactive VE library for affect induction that uses standardised validation protocols for new materials and none that uses the dimensional model of affect recognition as its theoretical basis. This article gives a clear example about how this VE library could be built in a standardised manner by using online questionnaire surveys for the VE validation as an initial step before re-validation in laboratory-based environments. We argue that this initial step is time and cost-efficient. In this online survey the entire VE and its individual events/ objects/features can be evaluated in one step. The aim of this study was to validate purpose-built VE as part of the AVEL library that induced different valence and arousal states in an online study. For this, several interactive room-scale virtual scenarios were newly designed and developed, giving us full creative and experimental control over the properties of these VEs. These VEs also contained 3D spatial objects and interactive events aiming to stimulate different variations of valence and arousal intensities to viewers throughout the experience. The VEs were validated using an online survey. The survey included videos of the environments recorded from the point of view of a user in VR, as well as images of the events/objects that were presented in the VE (1 video and 14 event/object images per VE). Participants were asked to rate their perceived affect in terms of arousal and valence for the videos and for the event/object images. Mean valence and arousal ratings for the events/objects were also correlated with the memory accuracies levels for these events/objects. Additionally, presence levels (per VE condition) and the alexithymia scores were measured using short questionnaires.

The participants’ responses showed that VE videos induced the expected mean arousal and valence ratings in the office based neutral, positive and negative VE scenarios, confirming our primary hypothesis. More specifically, the neutral scenario elicited neutral valence and low arousal, while the positive and negative scenario elicited highly positive and highly negative valence respectively, and higher arousal ratings, which was found to be increased for the negative VE. These VE conditions also induced valence and arousal ratings that were significantly different from each other. Unfortunately, the baseline environment was rated as more positive and more arousing than the neutral environment, which was against our initial expectations. This prompted us to not use this VE for baseline recordings in future experiments.

Apart from the ratings per VE video, the valence and arousal ratings were also recorded for each event/object in the office–based VEs. This allowed a more fine-grained analysis of affect responses and to inspect the effect of the environmental properties in more detail, which is not possible with the one-point rating at the end of each VR scene or video often used in previous libraries [22, 23]. With this approach, we combined entire VE and object validations as used in recent 3D object libraries [24–26]. This enabled us to find and exclude those objects/events that did not consistently induce the desired affective responses. As expected, most (but not all) events/objects elicited the expected valence and arousal ratings, and interactive events were rated as more positively/negatively valenced and more arousing compared to VE-specific static objects (see S1 Table in S1 Data). This result was in accordance with our primary hypothesis. Although, comparisons between dynamic and static or pictorial stimuli have been explored mainly for specific targeted emotions such as fear [69] and 2D facial expression libraries in the past e.g. [70], our findings signify the importance of utilising interactive or dynamic stimuli in VEs when wanting to provoke emotionally intense responses from users, thus making it a critical step towards in the creation of engaging virtual experiences.

Regarding the relationship between affective intensity and memory, significant medium to strong correlations were found between memory accuracies, arousal and valence ratings. These results agree with the expected relationship between affect intensity and memory [40–43]. Recording memory accuracy can help to establish how salient events/objects are within a VE, how much they draw attention towards them to be then being memorised which reflects a deeper level of processing. For example. interactive events were more memorable than still objects. This method can be used as a potential tool to validate events/objects beyond their affective impact without strictly controlling for low level features (such as light, spatial contrast, etc.) which can be cumbersome. This study also investigated whether presence and affective ratings were correlated in this online study. Presence scores were recorded for each VE video presented outside VR settings in this online survey. As expected, the average level of presence was rated low due to the nature and type of the used content-presentation tool. Individual subscale scores were not compared between VEs. As expected, low values were observed for all subscales, esp. the realism subscale, because of the stimuli presentation medium, but also potentially to the nature of the events/objects chosen for each VE. For example, some of the event stimuli in the positive VE may appear to be less realistic than others. However, these presence ratings are still very useful because they give researchers a baseline comparison relative to later presentations of these VEs in 2D/3D passive laboratory-based settings and, importantly, relative to 3D interactive settings. Interestingly, we already found presence differences when comparing the three VE environments when using the online survey format. More specifically, the negative VE provoked increased feelings of presence compared to the other VE conditions. Moreover, there were significant correlations between presence and arousal ratings for both positive and negative VEs, which agrees with the theory on the relationship between presence and arousal [71]. This result also suggests that a VR designer may be able enhance the presence levels within a VE by utilising elements with high affective intensity.

Finally, individual differences in alexithymia in relation to the valence and arousal ratings for each VE were investigated. High alexithymia can cause difficulty in recognising and regulating one’s emotions and has been shown to have an influence on how physiological responses are elicited in an affect stimulating context. Against the expectations, alexithymia did not influence the affective ratings for the entire VE in this study. However, when specific events/objects were analysed people with high alexithymia had more neutral ratings for events/objects in the neutral and negative VE compared to the low alexithymia group. This means that alexithymia can affect the emotional ratings of a virtual environment which is more visible when fine-grained ratings of events/objects are used. Therefore, we would recommend to routinely measure alexithymia in future affective validation and VR studies.

In conclusion, this study presented a clear example how to design affective VEs comprising of a room-based environment filled with several affective 3D events/objects, and how to validate these VEs using an online survey in a cost- and time-effective way. This method will save laboratory resources and it can help to build an online library for interactive VEs across research groups. In this study, the validation was based on affective valence and arousal self-ratings for each VE and for each event/object within this VE. Valence and arousal are the essential dimensions for measuring affect [72] following the circumflex dimensional model [38], and are predominantly used in affect detection and classification studies [73]. Additional dimensions, such as dominance, power and expectancy [74] could be easily added to extend the dimensions affect complex in future studies, following the proposed method of validation. In addition, memory accuracy for all events/objects were recorded to evaluate the depth of processing of the VE and if its events / objects, and presence ratings were collected for the entire VE. We argue that albeit all affective and presence ratings are expected to be higher when used in an immersive set-up, acquiring affective ratings early on from on-screen survey could provide a clear indication of flaws in the design (e.g., events with ambivalent affective impact). Thus, design challenges can be easily rectified at early processing stages This affective library specifically designed for room-scale VR settings, is to our best of knowledge the first library involving 3D, interactive, virtual environments and individual events/objects which have been individually rated using arousal and valence scores. The library can be easily extended and shared as an open-resource by including other VEs from our research group or other researchers. Such libraries can facilitate well-controlled and adaptive designs and studies with enhanced interactivity that can be used for experimental behavioural and psychophysiological research and in wider research settings. We do hope that more research groups take a similar approach of VE validation in order to interpret their data more accurately before starting their in-person VR data collection.

## Supporting information

S1 Data (DOCX)

S1 File Description of the virtual environments. Detailed description of the four environments, the event-stimuli used and the spatial structure of the virtual space, as well as gaze-based event detection tracking developed as part of the AVEL library. https://doi.org/10.17605/OSF.IO/G4U6W.
(DOCX)

S2 File AVEL survey data. All data from the survey. https://doi.org/10.17605/OSF.IO/G4U6W.
(TXT)
