# Supplementary material for: A reliable and robust online validation method for creating a novel 3D Affective Virtual Environment and Event Library (AVEL)
Source: PLoS One. 2023 Apr 13;18(4):e0278065. doi: 10.1371/journal.pone.0278065 (PMC10101521; doi:10.1371/journal.pone.0278065)
Supplement: S1 Data — (DOCX) [file pone.0278065.s001.docx]

## Supporting information

2. S1 Fig8. Mean valence and arousal ratings for each stimulus in each of the VE conditions. A list of all stimuli including static objects and animated events is displayed on the right side of each figure. A: neutral VE condition. B: positive VE condition. C: negative VE condition.


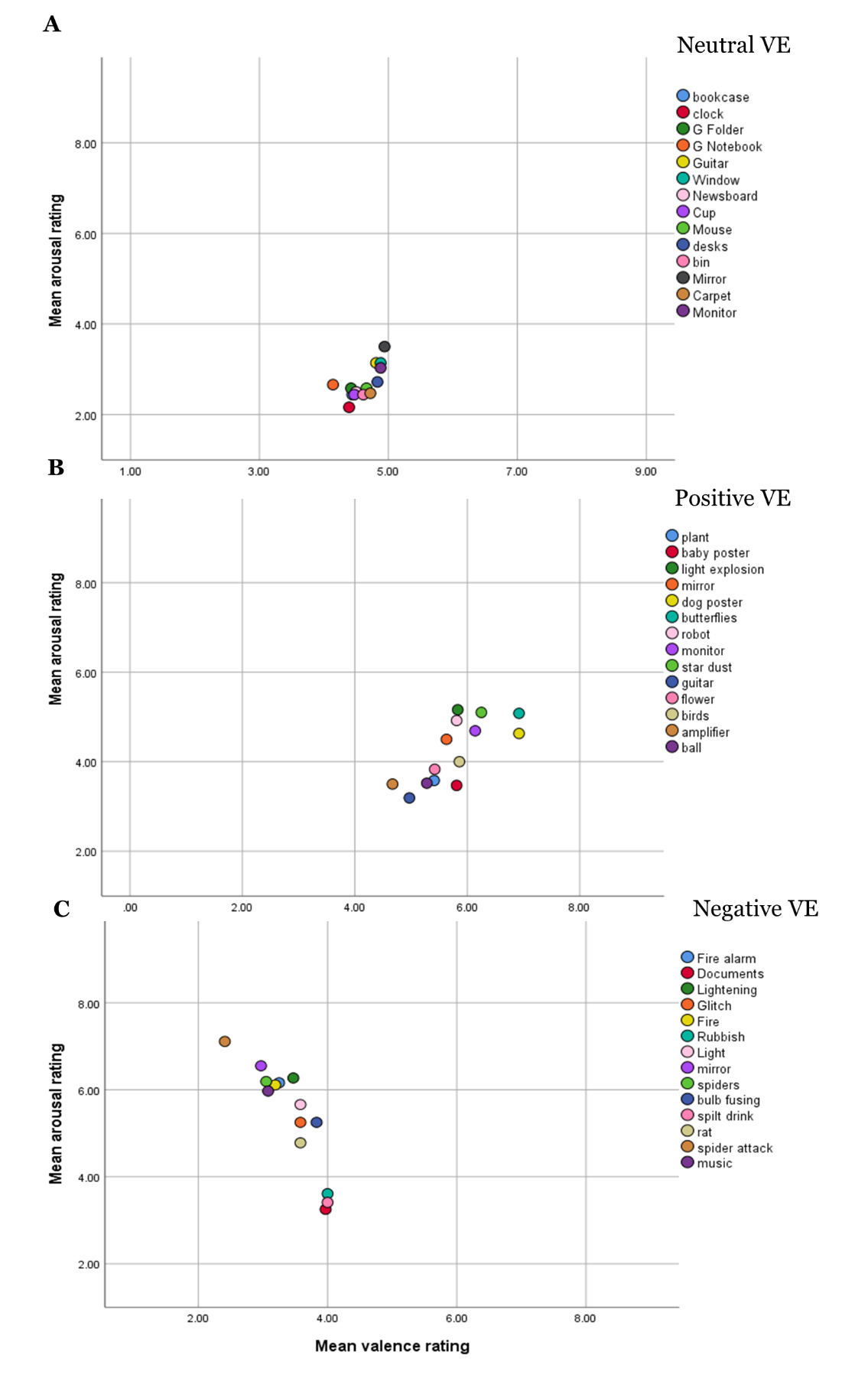


1. **S1 Table.** The average memory accuracy scores in percentages for each event are presented for each stimulus in each condition. The events per VE condition that scored the lowest on memory accuracy (lower 25%) and the events that scored the highest scores (upper 25%) are highlighted with pink and green colour respectfully. Interactive events are indicated with ‘^i^’.

| Neutral VE | | | | Negative VE | | | Positive VE | | |
| --- | --- | --- | --- | --- | --- | --- | --- | --- | --- |
| Stim.ID | M | Std. | Stim.ID | | M | Std. | Stim.ID | M | Std. |

| Bookcase | 91.05 | 28.55 | Fire Alarm ^i^ | 86.57 | 34.10 | Green Plant | 89.55 | 30.59 |
| --- | --- | --- | --- | --- | --- | --- | --- | --- |
| Clock | 61.19 | 48.73 | Documents | 22.39 | 41.68 | Baby Poster ^i^ | 70.15 | 45.76 |
| Green Folder | 55.22 | 49.73 | Lightening ^i^ | 91.05 | 28.55 | Light Explosion ^i^ | 88.06 | 32.43 |
| Grey Notebook | 41.79 | 49.32 | Glitch-View ^i^ | 55.22 | 49.73 | Mirror Reflection ^i^ | 85.08 | 35.63 |
| Guitar | 50.75 | 49.99 | Fire ^i^ | 89.55 | 30.59 | Dog Poster | 88.06 | 32.43 |
| Window | 89.55 | 30.59 | Rubbish Bin | 26.87 | 44.33 | Butterflies ^i^ | 95.52 | 20.68 |
| News-board | 71.64 | 45.07 | Flickering Light ^i^ | 91.05 | 28.55 | Robot ^i^ | 95.52 | 20.68 |
| Cup | 35.82 | 47.95 | Mirror ^i^ | 94.03 | 23.69 | Monitor Message ^i^ | 88.06 | 32.43 |

| Mouse (PC) | 76.12 | 42.64 | Spiders ^i^ | 88.06 | 32.43 | Star Dust ^i^ | 83.58 | 37.04 |
| --- | --- | --- | --- | --- | --- | --- | --- | --- |

| Desks | 97.02 | 17.02 | Light Bulb ^i^ | 74.63 | 43.52 | Guitar | 44.78 | 49.73 |
| --- | --- | --- | --- | --- | --- | --- | --- | --- |
| Bin | 55.22 | 49.73 | Cup (Spilt Drink) | 23.88 | 42.64 | Flower ^i^ | 52.24 | 49.95 |

| Mirror | 83.58 | 37.04 | Rat ^i^ | 41.79 | 49.32 | Birds ^i^ | 76.12 | 42.64 |
| --- | --- | --- | --- | --- | --- | --- | --- | --- |

| Carpet | 92.54 | 26.28 | Spider Attack ^i^ | 97.02 | 17.02 | | Amplifier | | 55.22 | | | 49.73 | |
| --- | --- | --- | --- | --- | --- | --- | --- | --- | --- | --- | --- | --- | --- |
| Monitor | 94.03 | 23.69 | Spooky Music ^i^ | 79.10 | 40.66 | | Ball | | 56.72 | | | 49.55 | |
|  |  | TOP 25% | | | |  | | **BOTTOM 25%** | |  |  | |  |
