# Supplementary material for: A reliable and robust online validation method for creating a novel 3D Affective Virtual Environment and Event Library (AVEL)
Source: PLoS One. 2023 Apr 13;18(4):e0278065. doi: 10.1371/journal.pone.0278065 (PMC10101521; doi:10.1371/journal.pone.0278065)
Supplement: S1 File — Detailed description of the four environments, the event-stimuli used and the spatial structure of the virtual space, as well as gaze-based event detection tracking developed as part of the AVEL library. https://doi.org/10.17605/OSF.IO/G4U6W. (DOCX) [file pone.0278065.s002.docx]

1. Description of the Virtual Environment

In this section, the design of four 3-D VEs is described. The environments were named ‘Baseline-relaxation scene’, ‘Neutral scene’, Positive scene’ and Negative scene’. The software used for the development were Unity3D game engine [1] to design the VE scenarios, and Autodesk Maya 3D [2] for the design of the 3D objects.

The first VE was a **baseline-relaxation scene** using a 360^o^ underwater environment and a smooth water audio-track (Figure 1). Participants of the main study would be entering this scene before each VE, for at least 2 minutes per visit. This environment was designed to allow the participant to relax before entering one of the main affective scenes, thus decreasing the physiological arousal which could be elevated due to the novelty of the media experienced [3]. The idea for the introduction of the water element of this VE was inspired by research on the restorative effects of aquatic environments [4,5]. We expected this environment to evoke low arousal and neutral/slightly positive valence levels. The addition of aquatic elements, and marine biota was avoided in order to control for high positive valence ratings [6].


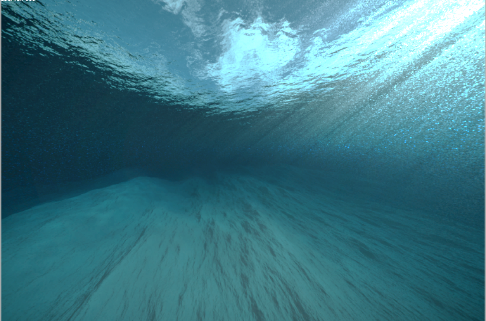


**Figure 1.** Screenshot from within the VE used for relaxation and baseline recording in pre-study survey.

The **remaining three VEs** were based on an existing office space (see photos of details of the actual room used in Appendix C Appendix*:* Study Materials’[7]). The VEs were mapped according to that space and populated with virtual counterparts of the physical objects. The virtual office room was 2.3m width x 2.90m length x 2.20m height with an allocated walking area of 1.6m x 2m which was consistent between all VEs. The dimensions and the basic synthesis of the space in those VEs was kept identical. In all settings, the room contained a bookcase, two office desks with chairs, a window, lights, two PCs with monitors, a small cupboard with a printer, a garbage bin, a mirror and two paper notebooks. The individual configurations for each VE were adjusted to evoke specific affective responses. In the positive VE and the negative VE, several parameters were altered to evoke either positive or negative affective responses, based on the low-level visual modifications and the integration of static and interactive objects (see Figure 2). The neutral VE was created to evoke a neutral mood with low arousal levels. These configurations are discussed per VE next.

**Figure 2.** Screenshots taken of the three affective VES from the user’s view (a, left side) and top view (b, right side). The first one (1a & 1b, top) is from the neutral environment, the second (2a & 2b, middle) is from the negative one and the third (3a &3b, bottom) is the positive VE. The square areas outlined in the top views (1b,2b,3b) show the perimeter of the walking area of the user.


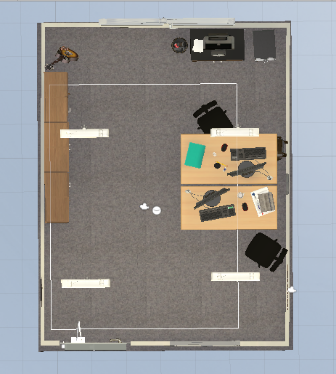

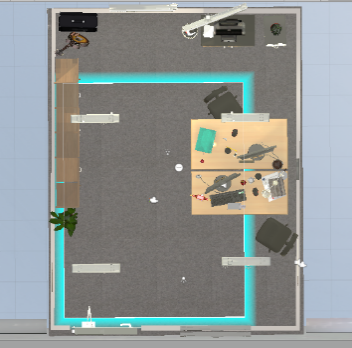

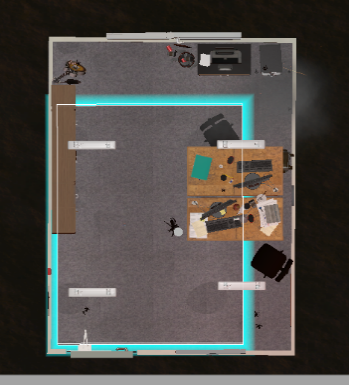


2b

2b

2b

2b

3b

3b

3b

3b


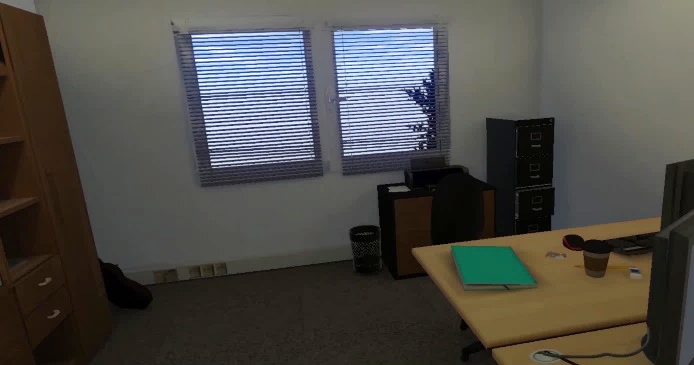

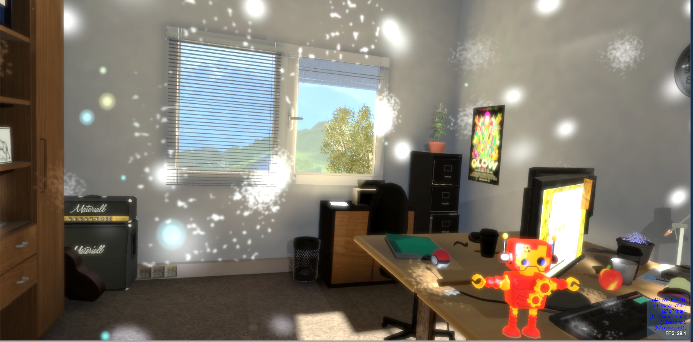

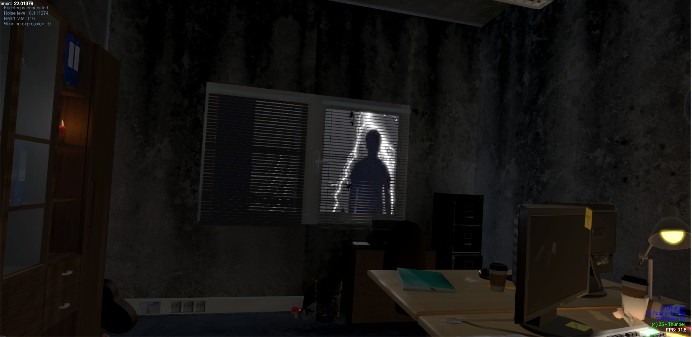


1a

1a

1a

1a

2a

2a

2a

2a

3a

3a

3a

3a

1b

1b

1b

1b

**Neutral VE.** The neutral environment contained all static basic objects of the office synthesis without the elements that were designed for negative or positive valence elicitation. The colour palette and temperature was kept in grey and cold faded tones, with low contrast to reduce the possibility for increased physiological arousal [8,9]. No audio samples or interactive events were planned for this VE. The lighting conditions were soft and dimmed with smooth shadows. This way the room was not strongly lighted or bright but completely visible for exploration by the user. A roman blind was designed in front of the window, to reduce the incoming light from the virtual sun embedded in the scene. The space outside the room, visible through the window was set to a grey, cloudy view with faded colour detail.

**Negative VE.** The colour palette for this room was set to intense, dramatic contrasts. The overall atmosphere was inspired from two scary/horror games in VR: ‘Resident Evil 7 VR’ and ‘Affected: the Manor’ [10,11]. The main lights were direct, switching on and off as if they were faulty using a custom-built switch with short, randomised time intervals. The walls and floor were covered with an additional material resembling of dirty, unpainted concrete. The synthesis of lights and textures was based on the design experience of the research team. Multiple additional objects were placed inside the room, including interactive and animated spiders, stressful notes, a ghost-like figure in the mirror, an animated shadow-figure appearing outside the window, an animated rat, a candle, litter placed around the bin, a fire trigger and an alarm. The majority of those events were triggered by the gaze of the user, e.g., a shadow outside the window or a ghost face in the mirror. Others like the ‘spider attack’ event was be triggered once, attempting to jump-scare the user. The rest of the spiders were activated throughout the whole VE experience by slowly follow the users gaze in the room, climbing on walls and main virtual furniture. Once the user had spent 65 seconds in the scene, a fire was triggered, and 10 seconds later the fire alarm went off requesting the user to head towards the exit and leave the room. The fire alarm included a bright, red light circling around the room in quick intervals and a loud siren. These events were designed to increase the physiological arousal towards the end of the experience and intensify the negativity of valence. Audio were incorporated in all interactive objects, including light bulbs, the ghost event, the shadow (lightning), the rat, the fire alarm, and the spiders. Some of audio were downloaded from the free sound audio library [12] .

**Positive VE.** Similarly, all audio-visual parameters were set to provoke pleasant feelings with variations of arousal for the positive environment. Multicolour synthesis was selected, including bright tones with intense colour hues and saturation. All lights were set brighter across the whole room and an intense sun light was designed to enter through the window. The roman blinds were not covering the view anymore allowing for the user to look outside the window. Along with static objects including posters, post-its, an apple, and pictures, multiple interactive events were programmed to be activated in different time ranges based on the gaze of the user. These interactive events were: a flock of butterflies entering the room through the window, birds flying outside the window, fairy lights or star dust inside the room, a robot dancing on the table, webcam feed of the user on the mirror, flowers growing, a plant moving and an interactive videos of a goat on a picture screen. Again, audio was incorporated in all interactive events and animated objects. Approximately after 75 seconds in the room, and if the user was not engaged with another event/object, the windows would open allowing laughter sounds to enter the room.

- 1. Affective stimuli: Interactive and static events

Table 1 shows the list of events and objects per scene used in the main study. Screenshots of all objects and events are available in Appendix C. Videos of the rooms and videos/pictures of the events were sent for evaluation via an online survey to participants (section 5.3). In this section, the overall interaction design and how the events can be triggered within the VEs will be described.

**Table 1**. List of objects and events for each virtual environment.

|  | Negative | Neutral |  | Positive |
| --- | --- | --- | --- | --- |
| 1 | Fire Alarm | Bookcase |  | Green plant |
| 2 | Documents | Clock |  | Baby poster |
| 3 | Window -Lightening/silhouette | Green Notebook |  | Light explosion |
| 4 | Glitch in viewpoint | Grey Notebook |  | Reflection in mirror |
| 5 | Fire | Guitar |  | Dog poster |
| 6 | Overflowing bin | Window |  | Butterflies |
| 7 | Flickering light (bulb fusing) | News board |  | Robot |
| 8 | Spooky mirror | Calendar/Cup |  | Monitor message |
| 9 | Spiders in room | Computer Mouse |  | Stardust (Light particles) |
| 10 | Light (bulb exploding) | Desks |  | Guitar |
| 11 | Spilt drink (cup) | Bin |  | Flower |
| 12 | Rat | Mirror |  | Birds |
| 13 | Spider attack | Carpet Floor |  | Amplifier |
| 14 | Spooky music | Monitor |  | Beach ball |
| 15 | Candle/skull* |  |  | Goat picture* |
| 16 | Office room* |  |  | Backpack* |
| 17 |  |  |  | Pokemon ball* |
| 18 |  |  |  | Office room* |
| 19 |  |  |  | Window (mountains)* |

| *Items marked with* (**) were added later on and thus were not included in the survey* |
| --- |

Every detail in the 3D VE room was designed and positioned manually Hence, all objects and their features (such as animations) can be controlled by the programmer. Current available VR experiences with tasks like games or creative applications like painting in 3D, allow the user to interact with 3D elements and control them in real-time. Similarly, in this study, we designed the VEs to allow free-walk exploration in 3D rooms, as a more natural, innate way of environment exploration rather than using controllers and joysticks. This ‘naturalistic’ way of navigation the virtual space could promote and enhance increased feelings of immersion and presence. As the affective stimuli were positioned in context at different locations of the room, and since the users’ gaze is completely dependent on their own body movements, stimuli were activated when visible to the user or when the user is directly looking at them.

For this reason, all objects and features (together we call them ‘events’) had an ‘interactive marker’ which was activated when once of the following conditions are met: (a) the gaze of the user is directed towards the object, and the fixation duration is larger than 2 seconds, or (b) the time passed since the start of the VE experience (based on a predefined sequence of event activation per VE). Once the pre-defined conditions for each stimulus were satisfied, an interactive marker was added with an ‘ID’, a number corresponding to the activated event. Up to three events could be active at the same time, including two events activated separately by the user’s gaze and one event activated by time passed. The event markers allowed us to also track the interaction periods on the data stream, by exporting the timestamps of the activated events together with the rest of the recorded data (physiological signals and movement data).

- 1. The task and area of interaction

Figure 3 shows an aerial view of the virtual office. The starting position of user was the same across all VEs (point A) looking towards the other end of the room (point B.) The area was designed to allow the user to approach certain areas and interactive objects while also avoid some others. For example, starting point A, the user could look to the right and approach the chair and the mirror on the wall, and would avoid walking onto the virtual tables as they would have perceived as physical obstacles. The desks were replicas of the actual desk of the experimenter, which also placed in the same area in the physical world. The idea behind it was to make the overall experience more believable by giving some of the expected proprietary haptic feedback. For example, when the user could touch the virtual desk, she could actually feel the real physical desk.


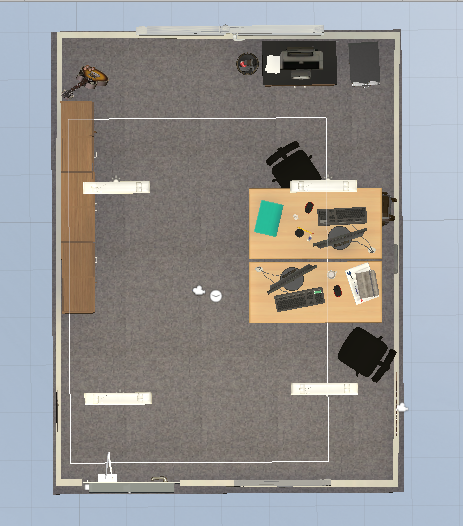


A

B


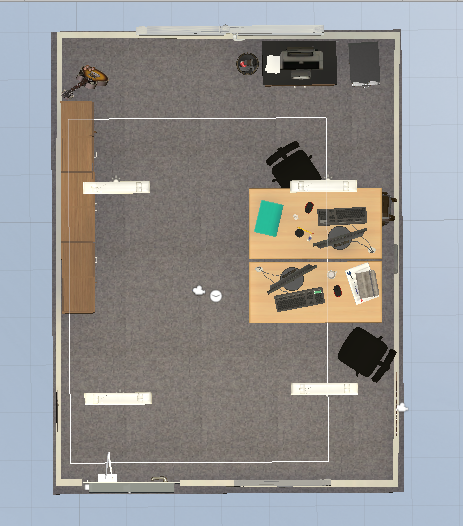


A

B

**Figure 3.** Top view of the scene used for the VEs. The write rectangle shows the perimeter of the user's walking area. The user would start from point A (left figure) then to point B while exploring the room, and back to point A (right figure) to exit the scene.

- 1. Gaze ray-casting for event marking

In order to find the objects that are in the user’s point of view in real time, an invisible ray was casted from the middle of the user’s point of view, facing forwards. The function ‘*Raycast*’ from the ‘*Physics*’ library in Unity Engine was used. Since the room was overall small and the field of view set at 60^o^ wide, there were only up to 4 different interactive events visible at the same time. We prioritised the objects/events that were in closer proximity to the user’s position and the ones that were actually visible to the user, i.e. not hidden behind another object such as a monitor hiding the poster on the wall or the flowerpot on the desk (see example Figure 4). Figure 5 shows the ray-casting in real time within the positive VE. In this figure, the camera icon is the viewpoint of the user looking towards the virtual robot. In this particular area of the scene the affective, interactive events were the robot and the monitor. All other objects used as decorative contextual props were categorised as static objects, e.g., apple, mouse, pencil, coffee-cup. As seen in Figure 5 and Figure 6 all these objects and events were currently in the user’s field of view. For demonstration, ray-casting lines are displayed in yellow for neutral objects and in red for interactive.


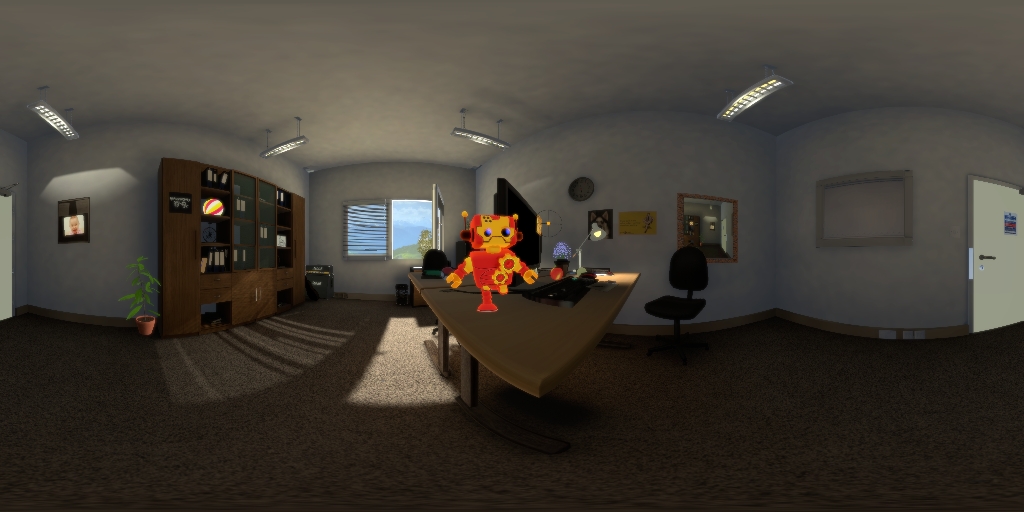


**Figure 4.** 360 view of the VE. The yellow box (middle) shows the area that was visible to the user.


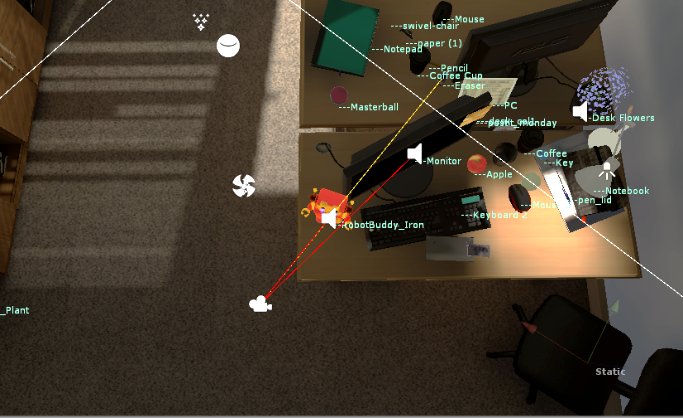

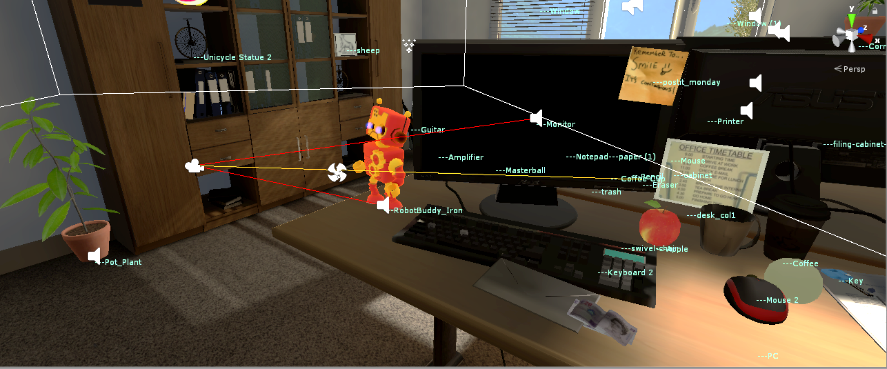


**Figure 5.** Side and top view of the ray-casting from the viewpoint of the user towards objects in the VE. The rays are displayed in yellow for static neutral objects and in red for interactive events.


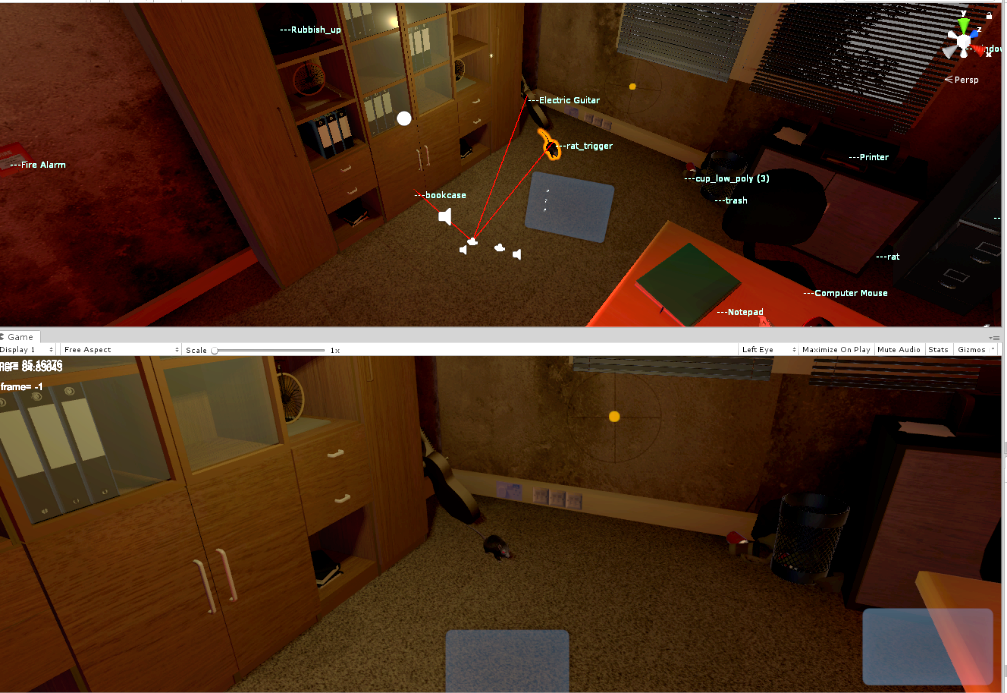

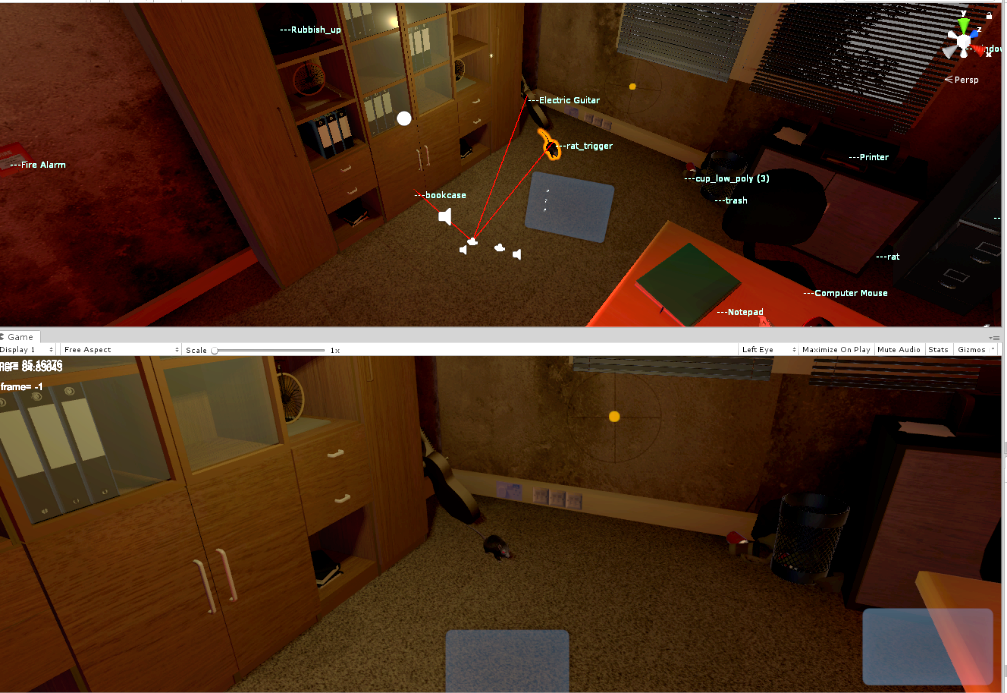


**Figure 6.** Left side: Experimenter view of the scene. Right side: User view of the scene.

Once the algorithm detected one or more event-markers, it activated the corresponding event*(*s). For example, looking at the robot activated a specific audio clip and the robot started dancing based on a predefined animation. The animation and the audio clip only stayed activated if the user’s gaze continued to look at the robot. Otherwise, it would automatically freeze. In addition, all surrounding visible ‘static’ objects were registered as ‘visible objects’ in the system and were also exported with timestamps in separate list (ASCII format).

Unlike the static objects, all events were designed to be visible to each participant for at least one time throughout the VR experience. For this purpose, certain conditions were designed, which were applied according to the nature of the stimuli-events. Short events (e.g., email on the monitor, in Positive VE) were able to replicate their animation up to 5 times once the gaze-ray was directed at them for longer than 2 seconds. While longer events (such as butterflies or fire) would only happen once, following a pre-defined activation time (i.e., >60 seconds in the VE). All interactive objects and events were enriched with audio clips which especially in the case of the longer events, attracted the attention and the gaze of the user towards the direction of the event or object. In the case where two interactive events were active at the same time, both their audio clips would be active at the same time. The volume of the audio clips was programmed to change in relation to the user’s gaze and position, relative to the wave source. Audio clips from interactive objects would fade out and stop when the user would stop interacting with the corresponding object-event. Some more elaborate events, like fire in the negative VE, triggered a sequence of other abstract stimuli (non-interactive) such as the fire sound, smoke in the room, and the fire alarm with the red light. These events were all timed to be activated within 15 seconds from the activation of the event ‘fire’. Of those only the ‘fire’ event and the ‘fire alarm’ had individual event markers.

- 1. Exiting the scene

Exiting a VE could be executed either manually by the experimenter or by the participant, via the participant’s direct interaction with the virtual door in the office-based VEs. Specifically, the participant could go towards the virtual door and ‘touch’ it with the controller, see Figure 46. The door would only open when the participant had spent at least 70 seconds in the VE. Once the participant exited a room, they would return to the ‘baseline VE’.


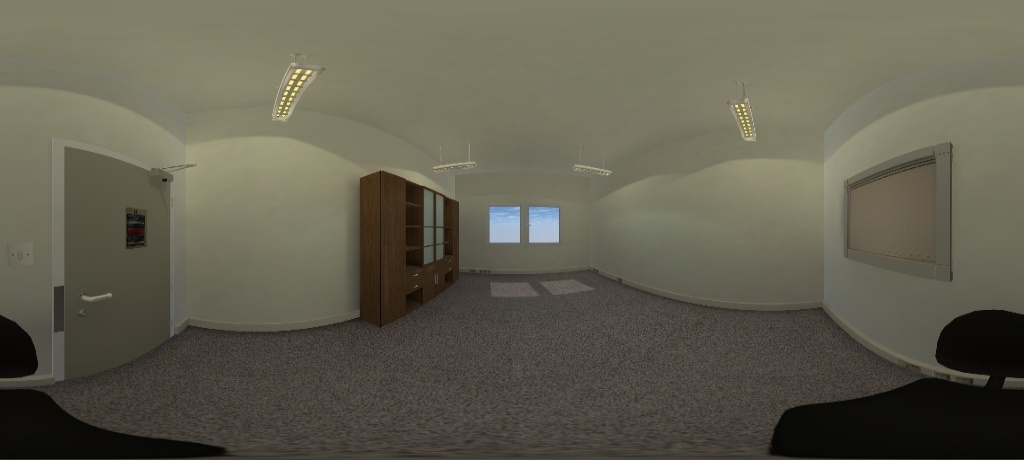


**Figure 7.** 360^o^ view of an office-based VE. The virtual door (indicated by the orange outline, left) was interactive and could assist participants exiting a VE by ‘touching’ it with the use of the hand controller.

**References**

1. Unity Real-Time Development Platform | 3D, 2D VR & AR Visualizations. [cited 15 Oct 2019]. Available: https://unity.com/

2. Free Software for Students & Educators | Maya | Autodesk. [cited 3 Jan 2020]. Available: https://www.autodesk.com/education/free-software/maya

3. Bradley MM, Lang PJ, Cuthbert BN. Emotion, novelty, and the startle reflex: Habituation in humans. Behav Neurosci. 1993;107: 970–980. doi:10.1037/0735-7044.107.6.970

4. White M, Smith A, Humphryes K, Pahl S, Snelling D, Depledge M. Blue space: The importance of water for preference, affect, and restorativeness ratings of natural and built scenes. J Environ Psychol. 2010;30: 482–493. doi:10.1016/j.jenvp.2010.04.004

5. Thoma MV, Mewes R, Nater UM. Preliminary evidence: The stress-reducing effect of listening to water sounds depends on somatic complaints. Med (United States). 2018;97. doi:10.1097/MD.0000000000009851

6. Cracknell D, White MP, Pahl S, Nichols WJ, Depledge MH. Marine Biota and Psychological Well-Being: A Preliminary Examination of Dose-Response Effects in an Aquarium Setting. Environ Behav. 2016;48: 1242–1269. doi:10.1177/0013916515597512

7. Mavridou I, Balaguer-Ballester E, Seiss E, Nduka C. Affective State Recognition in Virtual Reality from Electromyography and Photoplethysmography using Head-mounted Wearable Sensors. Bournemouth Univeristy. 2021. Available: http://eprints.bournemouth.ac.uk/35917/

8. Noguchi H, Sakaguchi T. Effect of Illuminance and Color Temperature on Lowering of Physiological Activity. Appl Hum Sci J Physiol Anthropol (Appl Hum Sci. 1999.

9. Marín-Morales J, Higuera-Trujillo JL, Greco A, Guixeres J, Llinares C, Scilingo EP, et al. Affective computing in virtual reality: emotion recognition from brain and heartbeat dynamics using wearable sensors. Sci Rep. 2018;8. doi:10.1038/s41598-018-32063-4

10. CAPCOM：RESIDENT EVIL 7 biohazard. [cited 3 Jan 2020]. Available: http://residentevil7.com/uk/#_top

11. AFFECTED - The Manor on Gear VR | Oculus. [cited 3 Jan 2020]. Available: https://www.oculus.com/experiences/gear-vr/1058194390920956/

12. Freesound - Freesound. [cited 28 Jan 2020]. Available: https://freesound.org/
